# Effect of weight‐loss diets prior to elective surgery on postoperative outcomes in obesity: A systematic review and meta‐analysis

**DOI:** 10.1111/cob.12485

**Published:** 2021-08-31

**Authors:** Natalie Pavlovic, Robert A. Boland, Bernadette Brady, Furkan Genel, Ian A. Harris, Victoria M. Flood, Justine M. Naylor

**Affiliations:** ^1^ South Western Sydney Clinical School University of New South Wales Sydney New South Wales Australia; ^2^ Fairfield Hospital South Western Sydney Local Health District Sydney New South Wales Australia; ^3^ Discipline of Physiotherapy, Sydney School of Health Sciences, Faculty of Medicine and Health The University of Sydney Sydney New South Wales Australia; ^4^ Western Sydney University School of Science and Health Sydney New South Wales Australia; ^5^ Liverpool Hospital South Western Sydney Local Health District Sydney New South Wales Australia; ^6^ St George and Sutherland Clinical School University of New South Wales Sydney New South Wales Australia; ^7^ Whitlam Orthopaedic Research Centre, Ingham Institute South Western Sydney Local Health District Sydney New South Wales Australia; ^8^ Sydney School of Health Sciences, Faculty of Medicine and Health The University of Sydney Sydney New South Wales Australia; ^9^ Westmead Hospital Western Sydney Local Health District Sydney New South Wales Australia

**Keywords:** obesity, postoperative complication, surgery, weight‐loss diet

## Abstract

This systematic review investigated the effects of weight‐loss diets before elective surgery on preoperative weight loss and postoperative outcomes in people with obesity. Electronic databases were searched from inception to May 2021. Inclusion criteria were prospective cohort or randomised controlled studies that compared effects of weight‐loss diets to standard care on postoperative outcomes in adults with obesity awaiting surgery. Participants with cancer or undergoing bariatric surgery were excluded. Data on preoperative weight change, length of stay, postoperative complications and patient‐reported outcome measures were extracted and synthesised in meta‐analyses. One randomised controlled trial involving total knee arthroplasty and two that investigated general surgery were eligible that included 173 participants overall. Each study compared low‐calorie diets using meal replacement formulas to usual care. There is very‐low‐quality evidence of a statistically significant difference favouring the intervention for preoperative weight loss (mean difference [MD] −6.67 kg, 95% confidence interval [CI] −12.09 to −1.26 kg; *p* = 0.02) and low‐quality evidence that preoperative weight‐loss diets do not reduce postoperative complications to 30 days (odds ratio [OR] 0.34, 95% CI 0.08–1.42; *p* = 0.14) or length of stay (MD −3.72 h, 95% CI −10.76 to 3.32; *p* = 0.30). From the limited data that is of low quality, weight loss diets before elective surgery do not reduce postoperative complications.

**What is already known about this subject**

- The prevalence of obesity is increasing worldwide
- Obesity is associated with greater risk of postoperative complications.
- Minimal research has been conducted on the effect of diet‐based preoperative weight loss on postoperative outcomes.

**What this study adds**

- This review is currently the only systematic review in the English literature that has investigated the effects of diet‐based weight‐loss interventions in people with obesity undergoing elective surgery excluding bariatric surgery.
- Weight loss through diet interventions can be achieved before elective surgery.
- There is low‐quality evidence that weight‐loss diets do not reduce postoperative complications to 30 days.

## 1 INTRODUCTION

Worldwide, the rate of obesity has almost tripled since 1975 with over 650 million adults, or 13% of the population, having obesity in 2016.^1^ The rising incidence of obesity has resulted in more people classified with obesity (defined as a body mass index [BMI] of 30 kg/m^2^ or greater) before undergoing elective surgery. For example, in the United States, the rates of extreme obesity (BMI ≥40 kg/m^2^) among those undergoing knee arthroplasty have doubled from 1993 to 2003.^2^ Increasing obesity is problematic because people with obesity are at a greater risk of certain postoperative complications, with greater health and financial burdens to the patient and the health system.^3^ Specifically, obesity is associated with elevated risks for revision surgery,^4^ wound complications,^4^, ^5^, ^6^ venous thromboembolism,^6^ pulmonary emboli,^4^ urinary tract infection,^5^ with worse long‐term patient‐reported functional outcomes,^7^ reduced mobility^7^ and inadequate activity levels postoperatively; all of which increase risk of chronic disease. Consequently, health professionals recommend preoperative weight loss to improve postoperative outcomes. That said, obesity is not always associated with worse postoperative outcomes because for instance, lower mortality rates may occur among people with obesity when compared to people who are underweight.^8^ Thus, it remains unclear whether weight loss before surgery should be recommended.

Recently, some health services worldwide have restricted criteria for undergoing elective surgeries based on weight. For example, the National Health Service in England reported that 31% of Clinical Commissioning Groups, who are responsible for service provision, have at least one mandatory policy on BMI level and weight management before elective surgery.^9^ Similarly, the surgeons at Logan Hospital in Queensland, Australia declined to operate on people with obesity unless they lost 10% of their body weight.^10^ In this case, a dietitian‐led presurgical weight management programme was implemented for their patients to achieve this target. Weight‐loss diets are a safe option, with potential cost benefits to the individual and health‐care system. However, little is known about the evidence in support of weight‐loss diets before any elective surgery to decrease adverse events postoperatively in populations with obesity. Thus, the objective of this review is to determine the effect of preoperative weight‐loss diets on postoperative clinical and service outcomes in people with obesity undergoing elective surgery.

## 2 METHODS

This systematic review was performed using methods from the Cochrane Handbook for Systematic Reviews of Interventions^11^ and according to the preferred reporting items for systematic reviews and meta‐analysis (PRISMA) guidelines.^12^ It was registered prior to commencement on Open Science Framework in February 2020 (https://osf.io/dgf3t) and PROSPERO (CRD42020154074).

### 2.1 Inclusion criteria

Eligibility criteria for included studies are summarised in Table 1.

**TABLE 1:** Eligibility criteria for inclusion of studies

Parameter	Inclusion criteria	Exclusion criteria
Participants	Adults aged ≥18 years BMI ≥30 kg/m^2^ Awaiting elective surgery including (but not limited to): orthopaedic procedures, cardiac surgery and gastrointestinal surgeries	Bariatric or cancer‐related surgeries were excluded because postoperative outcomes can be confounded by unique postoperative complications associated with such procedures or the underlying condition
Intervention	Weight‐loss diets prior to surgery Including (but not limited to): dietary modification, caloric restriction, meal replacement	Pharmacological weight loss Exercise alone Bariatric surgery as the weight‐loss intervention prior to elective surgery Weight loss prior to bariatric surgery
Comparator	Eligible intervention comparators (control groups) included elective surgical waiting lists where participants underwent usual or standard care This may include receiving general advice about healthy eating provided by a preadmission clinic or GP	Control groups that prescribed specific preoperative weight‐loss interventions were excluded
Outcomes	Primary outcome Postoperative complications to 90 days Secondary outcomes Amount of weight loss Acute length of hospital stay Discharge destination Duration of inpatient rehabilitation Patient‐reported outcomes for pain, function and quality of life Time take to return to work in any capacity Time taken to return to full work duties	
Study design	Prospective studies including randomised controlled trials and non‐randomised controlled trials, cohort studies	Retrospective studies Studies retrospectively assessing registry data Studies published in languages other than English

### 2.2 Search strategy

A preliminary limited search of MEDLINE, CINAHL and Scopus databases was performed to identify relevant keywords contained in study titles, abstracts, and subject descriptors, and their synonyms to inform an extensive search strategy, informed by a librarian (Supplementary File 1). Electronic databases searched from inception to the 14th of May 2021 included: MEDLINE, EMBASE, Cochrane Database of Systematic Reviews (references from eligible reviews were reviewed for eligible studies), Cochrane Central Register of Controlled Trials (CENTRAL), CINAHL, Scopus, ClinicalTrials.gov and WHO International Clinical Trials Registry Platform (WHO ICTRP). There were no limits for the year of publication or publication status. Reference lists of included studies and grey literature were reviewed to identify additional studies.

### 2.3 Study selection

Search results were merged using EndNote and duplicate records were removed. Two reviewers (NP, RB) independently screened articles for relevance and excluded irrelevant articles based on titles and abstracts. Multiple reports of the same study were identified, with the most recent version included for review. Full texts of the remaining articles were independently assessed by NP and RB for inclusion against selection criteria. Disagreements were resolved through discussion with a third reviewer (JN).

### 2.4 Risk of bias assessment

Selected studies were independently assessed for methodological validity by two reviewers (NP, RB). The Cochrane Handbook's Risk of Bias (RoB) Version 2 checklist^13^ was used to assess individual outcomes from randomised controlled trials (RCTs) according to five domains of bias (randomisation process, deviations from intended interventions, missing outcome data, measurement of the outcome, and selection of the reported result). A priori, it was planned to use the Cochrane Handbook ROBINS‐I tool^14^ to assess the risk of bias in prospective non‐randomised trials. Discrepancies between reviewers were resolved through discussion. All studies, regardless of methodological quality, underwent data extraction and synthesis where possible.

### 2.5 Data extraction

Standardised items in an Excel spreadsheet were used by two independent reviewers (NP, RB) to record the following extracted data from eligible studies: title; author; year of publication; journal; study design; setting; participant characteristics; recruitment procedures utilised; trial size; preoperative weight‐loss intervention characteristics; details of the control; type of surgery; follow up or study duration; outcomes; outcome measurements; data analysis methods; details needed for risk of bias; author contact details; funding source. Data discrepancies were resolved through discussion. Three corresponding authors were contacted via email for further information and all responded. All analyses were performed on intention‐to‐treat data.

### 2.6 Assessment of heterogeneity

Clinical and methodological heterogeneity was assessed for diversity in participants, interventions, outcomes, study characteristics, and risk of bias for included studies to determine whether meta‐analysis was appropriate. Statistical heterogeneity within each meta‐analysis was assessed using the *I* ^2^ statistic.^15^ Due to the small number of studies eligible for inclusion in this review the decision was made to include all studies in the meta‐analysis regardless of the *I* ^2^ statistic so long as the studies were similar with respect to clinical and methodological characteristics.

### 2.7 Assessment of publication bias

Published reports were compared against trial protocols to evaluate potential for publication bias. While assessment of publication bias was planned, there were insufficient studies (<10 studies) to construct a funnel plot or perform Egger's regression test.

### 2.8 Data synthesis

Data were analysed using Review Manager version 5.4.1 software from the Cochrane Collaboration.^16^ Differences between dichotomous outcomes are presented as odds ratios (ORs) and continuous outcomes are reported as mean difference (MD). 95% confidence intervals were calculated for both dichotomous and continuous outcomes. Random‐effects meta‐analysis was performed to pool outcomes from studies with similar characteristics. Due to the anticipated diversity of clinical and methodological characteristics of included studies, and because the influence of obesity appeared to vary, effect size was not assumed to be the same. Sensitivity analysis was not performed due to the small number of studies included. A priori, subgroup analysis was planned to compare participants who lost weight to those who did not, regardless of group allocation, however necessary data were not available. For continuous outcomes, pooled mean differences were calculated using the inverse‐variance method. The Peto method was used for analysis of postoperative outcomes because it is the least biased and most powerful method for event rates below 1%.^15^ Data that could not be pooled are presented in tables with results summarised in text.

### 2.9 Assessing certainty in the findings

Two reviewers (NP, FG) applied the Grading of Recommendations Assessment, Development and Evaluation (GRADE) approach^17^ to evaluate the quality of evidence according to four levels (high, moderate, low, and very low) to quantify the degree of confidence in the reported results per outcome. A Summary of Findings (SoF) table was created using GRADEPro software (GRADEPro GDT 2015)^18^ (Table 2).

**TABLE 2:** GRADE summary of findings

Weight‐loss diets compared to usual care for patients with obesity awaiting elective surgery
Patient or population: patients with obesity awaiting elective surgery Setting: community, outpatient setting (preoperatively), hospital inpatient (during surgery) Intervention: weight‐loss diets Comparison: usual care

Outcomes	Anticipated absolute effects^a^ (95% CI)		Relative effect (95% CI)	No. of participants (studies)	Certainty of the evidence (GRADE)	Comments
	Risk with usual care	Risk with weight‐loss diets				
Postoperative complications to 30 days post‐surgery	78 per 1000	**28 per 1000** (7–107)	**OR 0.34** (0.08–1.42) (p=0.14)	156 (3 RCTs)	⊕⊕◯◯ LOW^b^ ^,^ ^c^	The evidence suggests that weight‐loss diets result in little to no difference in postoperative complications to 30 days post‐surgery
Preoperative weight change (%)	The mean preoperative weight change (%) was **−0.36**%	MD **6.07% lower** (10.65 lower to 1.48 lower) (p=0.009)	—	159 (3 RCTs)	⊕◯◯◯ VERY LOW^c^ ^,^ ^d^ ^,^ ^e^	Weight‐loss diets may result in greater preoperative weight change (%) than usual care but the evidence is very uncertain
Preoperative weight change (kg)	The mean preoperative weight change (kg) was **−0.58** kg	MD **6.67 kg lower** (12.09 lower to 1.26 lower) (p=0.02)	—	159 (3 RCTs)	⊕◯◯◯ VERY LOW^c^ ^,^ ^e^ ^,^ ^f^	Weight‐loss diets may reduce preoperative weight (kg) but the evidence is very uncertain
Length of hospital stay post‐surgery	The mean length of hospital stay post‐surgery was **41.9** h	MD **3.72 h lower** (10.76 lower to 3.32 higher) (p=0.30)	—	156 (3 RCTs)	⊕⊕◯◯ LOW^b^ ^,^ ^c^	The evidence suggests that weight‐loss diets result in little to no difference in length of hospital stay post‐surgery

*Note*: GRADE Working Group grades of evidence: High certainty: We are very confident that the true effect lies close to that of the estimate of the effect. Moderate certainty: We are moderately confident in the effect estimate: The true effect is likely to be close to the estimate of the effect, but there is a possibility that it is substantially different. Low certainty: Our confidence in the effect estimate is limited: The true effect may be substantially different from the estimate of the effect. Very low certainty: We have very little confidence in the effect estimate: The true effect is likely to be substantially different from the estimate of effect.

Abbreviations: CI, confidence interval; OR, odds ratio; MD, mean difference.

^a^ The risk in the intervention group (and its 95% confidence interval) is based on the assumed risk in the comparison group and the relative effect of the intervention (and its 95% CI).

^b^ Downgraded by one level due to high risk of bias in one study due to deviation from intended intervention and missing outcome data and some concerns in the selective reporting of outcomes in the remaining studies.

^c^ Downgraded by one level because the sample size does not meet the Optimal Information Size (OIS).

^d^ Downgraded by one level due to high risk of bias in one study due to deviation from intended intervention and missing outcome data.

^e^ Downgraded by one level due to inconsistency (high *I* ^2^).

^f^ Downgraded by one level due to high risk of bias in the largest study due to selective reporting of outcomes and high risk of bias in one study due to deviation from intended intervention and missing outcome data.

## 3 RESULTS

### 3.1 Study inclusion

**FIGURE 1:**
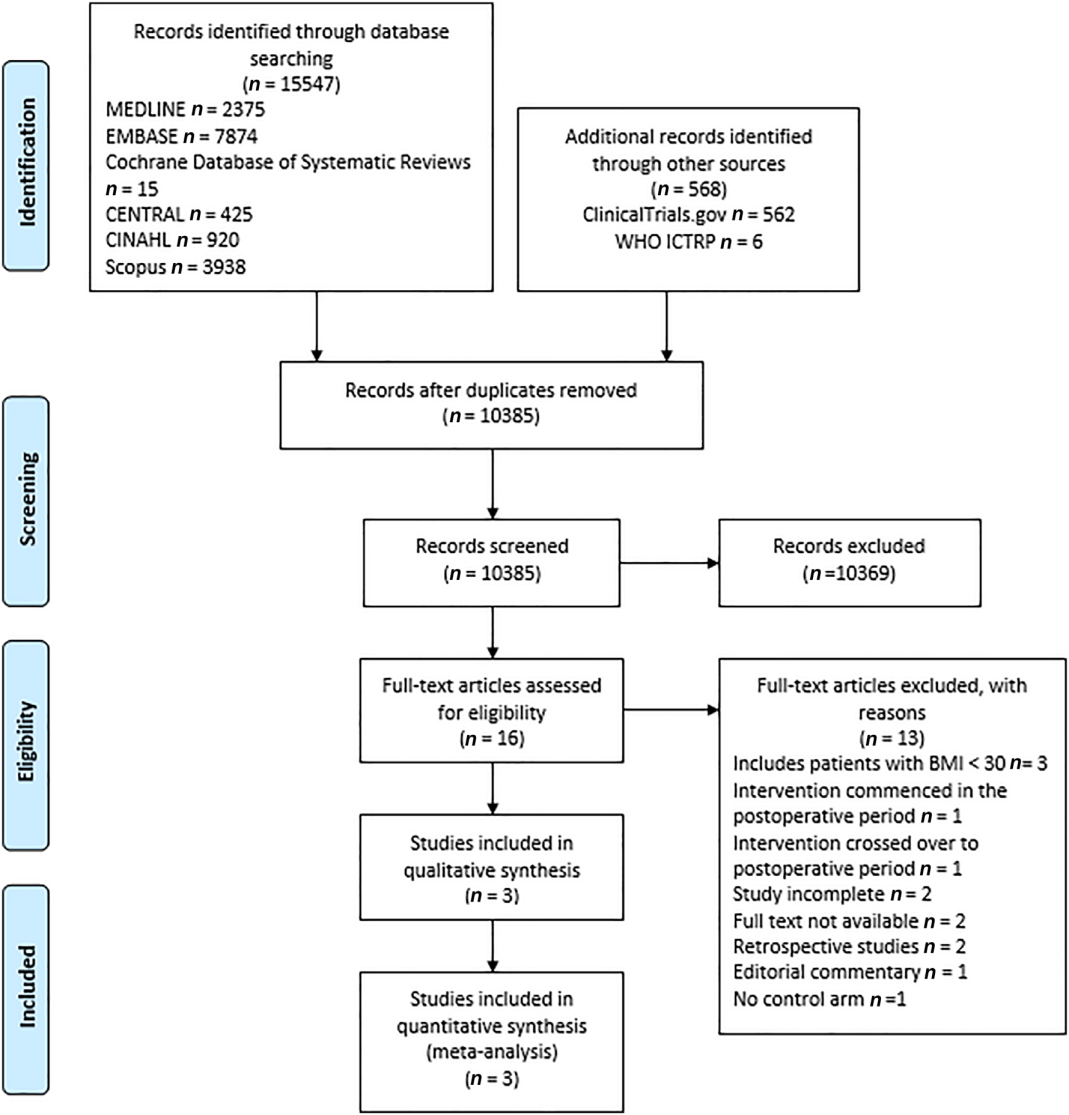
PRISMA flow diagram

A total of 15 547 references were retrieved through searches of electronic databases with another 568 references from other sources. After excluding duplicates and irrelevant articles from titles and abstracts, 16 articles remained (Figure 1). Thirteen articles were excluded (Table S1) because three^19^, ^20^, ^21^ included patients with BMI less than 30 kg/m^2^, one commenced the weight‐loss intervention in the postoperative period,^22^ one involved the intervention crossing over to the postoperative period,^23^ two were incomplete,^24^, ^25^ two were unpublished with no data available for inclusion,^26^, ^27^ two were retrospective studies,^28^, ^29^ one was an editorial commentary^30^ and one was an RCT with a weight‐loss diet in both the control and intervention groups.^31^ Three RCTs met the eligibility criteria.^32^, ^33^, ^34^ No prospective non‐RCTs were eligible for inclusion. No additional studies were identified by reference searching, yielding three articles that satisfied inclusion criteria and provided quantitative data for analysis.

### 3.2 Methodological quality

**FIGURE 2:**
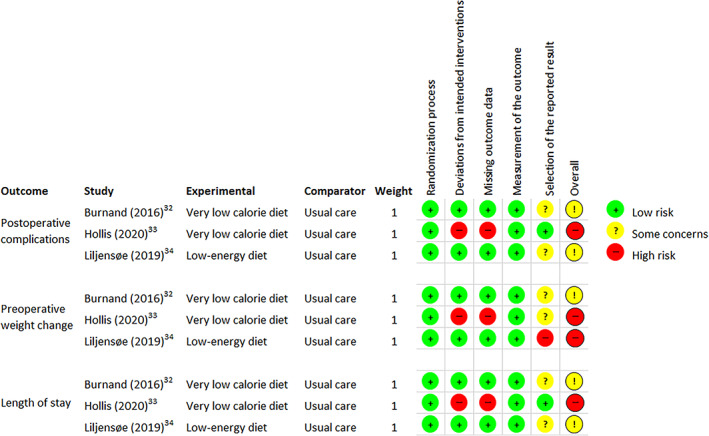
Risk of bias assessments for postoperative complications, preoperative weight change and length of hospital stay

Risk of bias assessments was completed for three outcomes: postoperative complications, preoperative weight change and length of stay (LOS) (Figure 2). One study demonstrated some concerns across all outcomes due to selective reporting of outcomes.^32^ Similarly, one study had some concerns for postoperative complications and LOS, and high risk of bias for preoperative weight change due to selective reporting of outcomes.^34^ One study demonstrated high risk of bias across all outcomes due to deviation from the intended intervention, missing outcome data and selective reporting of outcomes for preoperative weight change.^33^

Regarding the quality of the dietary interventions, no studies clearly stated a dietitian was involved in designing the intervention. Whilst all studies assessed dietary compliance with various methods, none utilised a validated tool to assess participant dietary compliance.

### 3.3 Characteristics of included studies

The three studies included were RCTs published in English. Two studies involved participants undergoing general surgery^32^, ^33^ and one examined participants undergoing total knee arthroplasty for knee osteoarthritis.^34^ A total of 173 participants were recruited with 82 and 87 participants randomised to control and intervention groups, respectively, aged from 21 to 85 years, with a baseline BMI greater than 30 kg/m^2^. Primary outcomes differed between studies; however, each study recorded postoperative complications to different time points and weight change from baseline. Characteristics of the included studies are presented in Tables 3 and 4. Each of the three studies implemented a weight loss diet involving very‐low‐calorie diets (VLCD) via meal replacement formulas, with differing calorie restrictions.

**TABLE 3:** Characteristics of included studies

Study	Burnand et al.^32^	Hollis et al.^33^	Liljensøe et al.^34^
Study design	RCT	RCT	RCT
Country	United Kingdom	Australia	Denmark
Journal	International Hepato‐Pancreato‐Biliary Association Inc.	Nutrition & Dietetics	Scandinavian Journal of Surgery
Age	21–69 years	Mean: 51.6 ± 13.1 years	46–85 years
Gender (*n*)	Male (4) Female (42)	Male (17) Female (29)	Male (22) Female (54)
Diagnosis (*n*)	Biliary colic (40) Cholecystitis (4) Obstructive jaundice (2)	Not specified	Knee osteoarthritis
Operation type (*n*)	Laparoscopic cholecystectomy	Laparoscopic cholecystectomy (27) Umbilical hernia repair (11) Ventral hernia repair (5) Inguinal hernia repair (3)	Total knee arthroplasty (TKR)
Baseline BMI (kg/m^2^), mean (SD)	Intervention: 34 (3.44) Control: 33.6 (3.35)	Intervention: 40.3 (6.0) Control: 40.7 (5.9)	Intervention: 31.6 95% CI (30.6–32.6) Control: 31.2 95% CI (29.8–32.6)
Trial size at baseline	46	50	77
Trial size at intervention	46	46	76
Intervention	Very‐low‐calorie diet	Very‐low‐calorie diet	Low energy liquid diet
Dietitian involved	No (advice only)	Yes	Yes
Control	Standard care. Dietitian available for advice only	Standard care	Standard care
Study duration	2 weeks preoperatively	8 weeks preoperatively 30 days postoperatively	8 weeks preoperatively 12 months postoperatively
Primary outcome	Intra‐operative time	Feasibility of implementing a preoperative very‐low‐calorie diet weight programme for patients with obesity awaiting general elective surgery.	Body weight (kg) Short‐form 36 subscale Physical Component Score (PCS)
Secondary outcome	Weight change Post‐op complications Length of stay (hours) Day‐case rates Perceived difficulty of procedure by surgeon	Surgical complications Weight (kg) Waist circumference (cm) Muscle mass (kg) Fat mass (kg) Quality of life (Impact of Weight on Quality of Life‐lite questionnaire) Intra‐operative time (min) Length of stay (days)	Short‐form 36 subscale Mental Component Score (MCS) Knee Injury and Osteoarthritis Outcome Score (KOOS) 6‐Minute walk test Fat mass (lean and bone) Bone mineral density Lipids Length of stay (days) Intra‐operative time Wound secretions at day 0 Blood pressure and heart rate

**TABLE 4:** Characteristics of weight‐loss diets from included studies

Study	Burnand et al.^32^	Hollis et al.^33^	Liljensøe et al.^34^
Intervention	Very‐low‐calorie diet	Very‐low‐calorie diet	Low‐energy liquid diet
Calorie restriction	Total caloric intake of 800 calories/day	700–800 calories/day with ≥0.75 g/kg adjusted body weight protein	Preoperative phase: 810 calories/day Postoperative maintenance phase: 1200 calories/day
Intervention details	Participants were given a diet sheet to follow	Optifast meal replacement shakes The consumption of three to four Optifast shakes mixed on water with an additional ≥two cups (non‐starch) vegetable or salad, at least 2 L of energy free fluids, and one teaspoon of vegetable oil were recommended daily for 8 weeks	Preoperative phase: Formula diet (Cambridge Weight Plan®, Northants, UK) consisting of ready‐to‐use meal, bars, and sachets to mix with water or skimmed milk (7.5 dL a day) to make shakes, soups, or porridge, consumed four times a day Nutritional education: weekly group sessions of 1.5 h led by an experienced dietitian for 8 weeks Maintenance phase: Regular meals combined with one formula diet serving per day. Eight group sessions led by the study dietitian
Duration	2 weeks preoperatively	8 weeks preoperatively	8 weeks preoperatively and 12 months postoperatively
Was a dietitian involved in administering the diet?	No. Dietitians were not directly involved but were available to provide advice as needed	Yes	Yes
Was a dietitian involved in the designing of the dietary intervention?	Yes. A member of the research team was a dietitian	Yes. The lead researcher is a senior dietitian	No information
Was there monitoring of adherence to the dietary intervention?	All patients were asked to complete a detailed diary survey for the 2 weeks prior to surgery	Adherence to the very‐low‐calorie diet programme was evaluated using the presence of urinary ketones in ≥50% of fortnightly samples collected by the clinic nurse over the 8‐week period	No information
Was a validated tool utilised to measure dietary adherence?	No information	No	No information

### 3.4 Review findings

The details for all primary and secondary outcomes are described in Tables S2 and S3. Meta‐analysis was undertaken where data were available as the included studies were similar with respect to participant and weight‐loss intervention characteristics and study design.

#### 3.4.1 Postoperative complications

**FIGURE 3:**
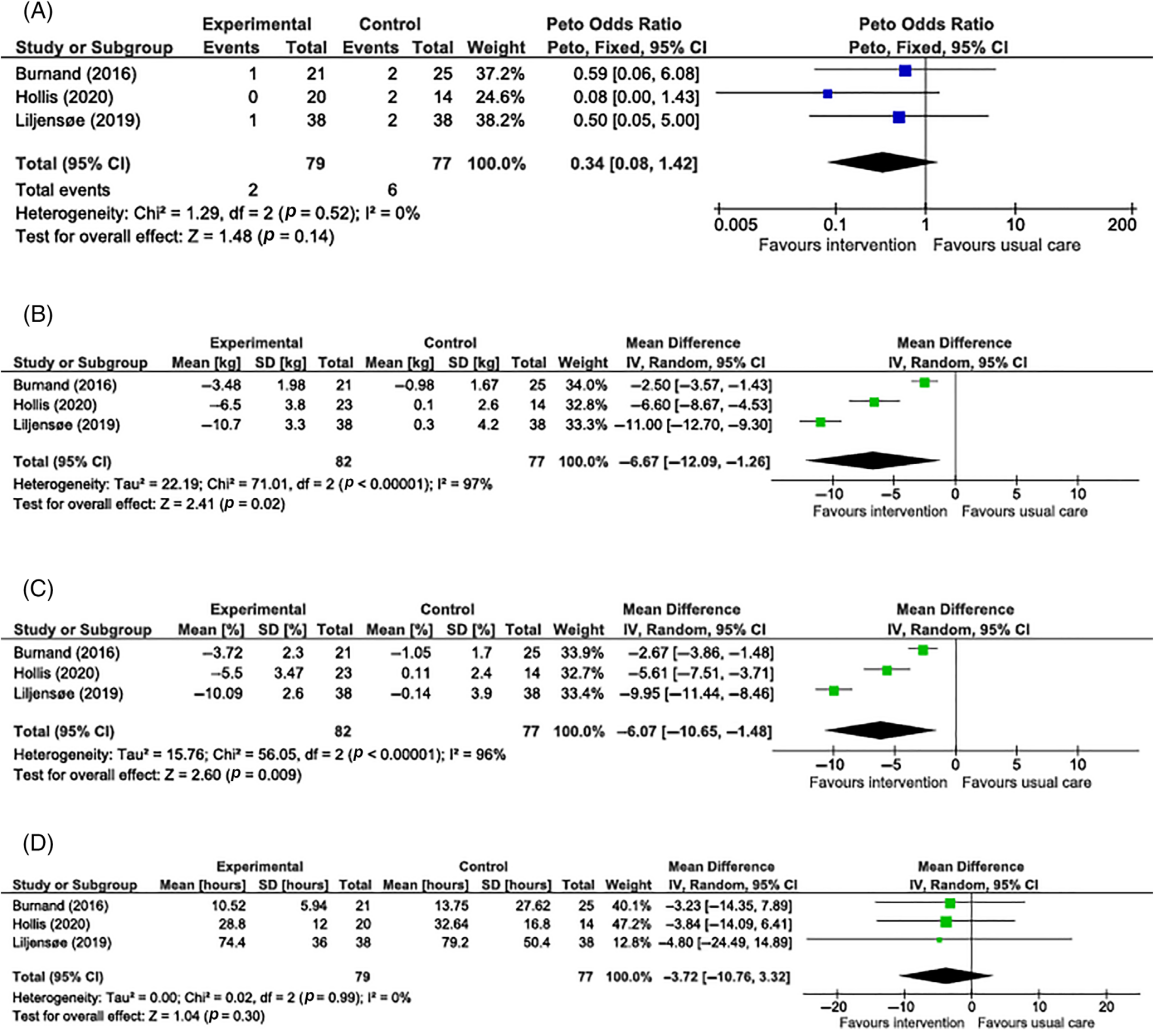
(A) Meta‐analysis of postoperative complications to 30 days post‐surgery when comparing preoperative diet‐based weight loss to usual care. (B) Meta‐analysis of preoperative weight change (kg) when comparing preoperative diet‐based weight loss to usual care. (C) Meta‐analysis of preoperative weight change (%) when comparing preoperative diet‐based weight loss to usual care. (D) Meta‐analysis of length of hospital stay (hours) when comparing preoperative diet‐based weight loss to usual care

The primary outcome, postoperative complications to 90 days, was reported in one study with a follow‐up period of 1‐year post‐surgery.^34^ Data for postoperative complications to 30 days was available from three studies with 156 participants. Random‐effects meta‐analysis did not show a statistically significant difference in postoperative complications to 30 days between intervention and control groups (OR 0.34, 95% confidence interval [CI] 0.08–1.42; *p* = 0.14) (*I* ^2^ = 0%, *p* = 0.52)(Figure 3A).

The following postoperative complications were routinely reported: surgical wound complications, wound secretion, wound infection and urinary tract infection. Other postoperative complications varied according to the procedure undertaken. Five arthroplasty‐related complications were recorded after the 90‐day postoperative period including manipulation under anaesthetic (one participant in each group), dislocation of the prosthesis without infection and two deep infections resulting in revision surgery (one participant from each group). One patient undergoing laparoscopic cholecystectomy had conversion to open surgery and one participant had a bile leak from an accessory duct requiring laparoscopy and washout. Readmissions occurred in two patients from one study, one from each group.

#### 3.4.2 Preoperative weight change

All studies reported mean change in weight from baseline to surgery in kilogrammes and as a percentage (159 participants). The pooled effect estimate showed very‐low‐quality evidence of a statistically significant difference favouring the intervention groups for preoperative weight loss in kilogrammes (MD −6.67 kg, 95% CI −12.09 to −1.26 kg; *p* = 0.02) (*I* ^2^ = 97%, *p* < 0.001) (Figure 3B) and as a percentage (MD −6.07%, 95% CI −10.65% to −1.48%; *p* = 0.009) (*I* ^2^ = 96%, *p* < 0.001) (Figure 3C). However, there was severe inconsistency for both outcomes from variation in between‐group differences in weight loss across studies.

#### 3.4.3 Length of stay

Acute hospital LOS was reported in all studies comprising data from 156 participants. The pooled effect estimate suggested that LOS was shorter in the intervention group, but the difference did not reach statistical significance: MD −3.72 h (95% CI −10.76 to 3.72; *p* = 0.30) (*I* ^2^ = 0%, *p* = 0.99) (Figure 3D).

#### 3.4.4 Patient‐reported outcome measures

Two studies collected patient‐reported outcome measures (PROMs). Liljensøe et al. reported no differences between groups from baseline to 1 year in Short‐Form 36 subscale Physical Component Score (1.3, 95% CI −2.2 to 4.7; *p* = 0.5), Short‐Form 36 subscale Mental Component Score (3.3, 95% CI −0.9 to 7.6; *p* = 0.1) and all Knee injury and Osteoarthritis Outcome Score subscales, including activities of daily living (2.8, 95% CI −5.8 to 11.4; *p* = 0.5), quality of life (8.3, 95% CI −3.4 to 20; *p* = 0.2), symptoms (4.9, 95% CI −3.1 to 12.9; *p* = 0.2), pain (0.8, 95% CI −9.0 to 10.5; *p* = 0.9) and sports/recreation (5.8, 95% CI −5.5 to 17.1; *p* = 0.3).^34^ Change scores were not available for the preoperative period. Hollis et al. reported improvements in health‐related quality of life measured by the Impact of Weight on Quality of Life Lite tool, compared to control (17, median range: −14 to 41.4; *p* = 0.009).^33^ However, only 15 and 4 complete pre‐post data sets were available for the intervention and control groups respectively, limiting the utility and value of this outcome. As such, a meta‐analysis for PROMs was not possible.

## 4 DISCUSSION

### 4.1 Summary of main findings

The primary objective was to determine whether weight‐loss diets in patients with obesity awaiting elective surgery improves postoperative outcomes. We found low‐quality evidence that preoperative weight‐loss diets do not reduce postoperative complications to 30 days and LOS when compared to a control group, although weight‐loss diets result in greater preoperative weight change than usual care.

A recent systematic review of RCTs, prospective and retrospective cohort studies evaluated the efficacy of preoperative weight loss through behavioural lifestyle changes following elective surgery in people with obesity.^35^ Preoperative weight‐loss interventions focussed on dietary changes, in particular, low‐calorie diets. The baseline mean BMI across included studies was significantly higher than in our review (BMI ≥40), likely due to the majority of studies focussing on participants undergoing bariatric surgery (with one study focussing on total knee and hip arthroplasty). Despite these differences, our findings are consistent with those conclusions that there were no significant differences in the rate of short‐term complications, despite weight loss favouring the intervention. The authors proposed that weight loss under 10 kg might not be enough to reduce perioperative risks in patients with obesity, or that the ‘obesity paradox’^36^, ^37^ protects against postoperative mortality and morbidity, whereby excess weight provides nutritional stores following surgery.^38^ Alternatively, BMI does not account for the proportion of lean muscle to adipose tissue, potentially resulting in fit, healthy individuals with low risk of postoperative complications classified with obesity due to muscle mass levels.^38^, ^39^ However, the follow‐up period was limited to 30 days, preventing conclusions about effects of weight loss on postoperative complications to 90 days. The authors were also unable to exclude the unique postoperative complications related to bariatric surgery because subgroup analyses according to the type of elective surgery were not performed.

### 4.2 Potential biases in the review process

This review has several strengths. First, it is relevant to the contemporary and growing rate of obesity globally, and the effects on postoperative patient outcomes and health‐care systems. Second, it was performed and reported according to the PRISMA guidelines described in the Cochrane Handbook of Systematic Reviews, per the pre‐registered protocol. Third, comprehensive assessment of evidence quality was conducted and incorporated GRADE. Finally, it only included RCTs and excluded retrospective studies.

Limitations of the current review included the limited generalisability of findings due to the small number of identified studies. Second, the effect of weight loss, as opposed to weight‐loss programmes, on postoperative outcomes could not be distinguished. Preoperative weight change data for each participant was unavailable for subgroup analysis which was planned to compare participants who lost weight to those who did not regardless of group allocation. Finally, each study utilised calorie restriction via meal replacement as the preoperative weight‐loss diets, limiting generalisability to other diet‐based interventions. However, such limitations reflect the dearth of investigations into dietary preoperative interventions for weight loss prior to elective surgery, particularly with respect to effects on postoperative complications.

### 4.3 Implications

Adequately powered and designed prospective studies are necessary to investigate preoperative non‐surgical and non‐pharmacological weight‐loss interventions as the proportion of people with obesity undergoing various surgeries is growing. Furthermore, preoperative weight change should be reported as a percentage to facilitate determining whether clinically significant weight loss is achieved, including details of body composition, with assessment of weight loss from lean versus adipose tissue. Finally, future research samples should be sufficiently large for subgroup analyses to compare participants achieving weight loss preoperatively to those who did not to determine effects on postoperative outcomes.

## 5 CONCLUSION

This review highlights the limited research into effects of weight‐loss diets on postoperative outcomes in people with obesity undergoing elective surgery that is not bariatric. The current findings provide low‐quality evidence that weight‐loss diets do not reduce postoperative complications to 30 days and LOS. No studies included in this review investigated weight‐loss diets other than meal replacements. Despite such limited evidence, health professionals continue to recommend weight loss before elective surgery for people with obesity. Further high‐quality and adequately powered prospective trials are needed to evaluate preoperative weight‐loss diets on postoperative outcomes.

## CONFLICTS OF INTEREST

No conflict of interest was declared.

## AUTHOR CONTRIBUTIONS

Natalie Pavlovic, Justine M. Naylor, Robert A. Boland, Bernadette Brady, Ian A. Harris and Victoria M. Flood were involved in the protocol development for the review. Natalie Pavlovic and Robert A. Boland were involved in the article screening, selection, data extraction and risk of bias assessment. Natalie Pavlovic was involved in data analysis. Natalie Pavlovic and Furkan Genel were involved in the formulation of GRADE assessment. Natalie Pavlovic, Justine M. Naylor, and Robert A. Boland are involved in monitoring the review progress. All authors contributed to the writing of the related manuscript. Justine M. Naylor is the guarantor of the review.

## Supporting information


**Data S1** Search strategies for each database for the systematic review and meta‐analysis.
**Table S1.** Excluded studies and reasons for exclusion.
**Table S2.** Postoperative complications.
**Table S3.** Outcome data.

## Data Availability

Data relevant to this systematic review are found in the article and supplementary file. Further data will be made upon request from the corresponding author.
