# Supplementary material for: Effect of weight‐loss diets prior to elective surgery on postoperative outcomes in obesity: A systematic review and meta‐analysis
Source: Clin Obes. 2021 Aug 31;11(6):e12485. doi: 10.1111/cob.12485 (PMC9286439; doi:10.1111/cob.12485)
Supplement: Supplementary file 1 — Data S1 Search strategies for each database for the systematic review and meta‐analysis. Table S1. Excluded studies and reasons for exclusion. Table S2. Postoperative complications. Table S3. Outcome data. [file COB-11-0-s001.docx]

**Effect of weight-loss diets prior to elective surgery on postoperative outcomes in obesity: a systematic review and meta-analysis**

**Authors:** Natalie Pavlovic, Robert A Boland, Bernadette Brady, Furkan Genel, Ian A Harris, Victoria M Flood, Justine M Naylor

**Corresponding Author:** Natalie Pavlovic (email: [Natalie.Pavlovic@health.nsw.gov.au](mailto:Natalie.Pavlovic@health.nsw.gov.au))

Fairfield Hospital, South Western Sydney Local Health District, Cnr Polding St & Prairievale Rd, Prairiewood, 2176, Sydney, NSW, Australia

**Supplementary Material**

**Supplementary File 1.** Search strategies for each database for the systematic review and meta-analysis.

**Supplementary Table 1.** Excluded studies and reasons for exclusion.

**Supplementary Table 2.** Postoperative complications.

**Supplementary Table 3.** Outcome data.

**Supplementary file 1. Search strategy for systematic review and meta-analysis**

**MEDLINE**

1. Elective Surgical Procedures/
2. Orthopedics/
3. Arthroplasty, Replacement/ or Arthroplasty, Replacement, Knee/ or Arthroplasty/ or Arthroplasty, Replacement, Hip/ or arthroplasty.mp.
4. Osteoarthritis/su [Surgery]
5. spinal fusion/ or laminectomy/
6. diskectomy/ or diskectomy, percutaneous/ or laminectomy/ or laminoplasty/
7. Thoracic Surgery/
8. Coronary Artery Bypass/
9. Laparoscopy/ or abdominal surgery.mp.
10. Digestive System Surgical Procedures/ or gastrointestinal surgery.mp.
11. Cholecystectomy, Laparoscopic/ or Cholecystectomy/
12. General Surgery/
13. 1 or 2 or 3 or 4 or 5 or 6 or 7 or 8 or 9 or 10 or 11 or 12
14. Body Mass Index/ or Obesity/ or Obesity, Morbid/ or Overweight/
15. weight loss.mp. or Weight Loss/
16. exp Diet/ or exp Diet Therapy/
17. meal replacement.mp. or Diet, Reducing/
18. Weight Reduction Programs/ or weight management.mp.
19. 15 or 16 or 17 or 18
20. "Length of Stay"/
21. Treatment Outcome/ or Postoperative Complications/ or surgical complications.mp.
22. 20 or 21
23. 13 and 14 and 19 and 22

“elective surgical procedures” OR “orthopedics” OR “replacement arthroplasty” OR “arthroplasty, replacement, knee” or “arthroplasty” or “arthroplasty, replacement, hip” or “arthroplasty” OR “osteoarthritis” OR “spinal fusion” OR “laminectomy” OR “diskectomy” OR “percutaneous diskectomy” OR “laminoplasty” OR “thoracic surgery” OR “coronary artery bypass” OR “laparoscopy” OR “abdominal surgery” OR “digestive system surgical procedures” OR “gastrointestinal surgery” OR “laparoscopic cholecystectomy” OR “cholecystectomy” OR “general surgery” AND “body mass index” OR “obesity” OR “morbid obesity” OR “overweight” AND “weight loss” OR “diet” OR “diet therapy” OR “meal replacement” OR “reducing diet” OR “weight reduction programs” OR “weight management” AND “length of stay” OR “treatment outcome” OR “postoperative complications” OR “surgical complications”

**MeSH terms**

Elective surgical procedures, orthopedics, “Arthroplasty, Replacement”, “Arthroplasty, Replacement, Knee”, Arthroplasty, “Arthroplasty, Replacement, Hip”, osteoarthritis, spinal fusion, laminectomy, diskectomy, percutaneous diskectomy, laminectomy, laminoplasty, thoracic surgery, coronary artery bypass, laparoscopy, digestive system surgical procedures, laparoscopic cholecystectomy, cholecystectomy, general surgery, body mass index, obesity, morbid obesity, overweight, weight loss, diet, diet therapy, reducing diet, weight reduction programs, length of stay, treatment outcome, postoperative complications

**Text word**

Arthroplasty, abdominal surgery, gastrointestinal surgery, weight loss, meal replacement, weight management, surgical complications

**EMBASE**

1. elective surgery/
2. orthopedic surgery/
3. revision arthroplasty/ or total knee arthroplasty/ or replacement arthroplasty/ or total arthroplasty/ or arthroplasty.mp. or hip arthroplasty/ or knee arthroplasty/ or arthroplasty/
4. ($knee replacement or $hip replacement).mp. [mp=title, abstract, heading word, drug trade name, original title, device manufacturer, drug manufacturer, device trade name, keyword, floating subheading word, candidate term word]
5. osteoarthritis/su [Surgery]
6. exp spine surgery/
7. exp heart surgery/
8. exp abdominal surgery/
9. laparoscopic cholecystectomy/ or cholecystectomy/
10. general surgery/
11. surgical patient/
12. 1 or 2 or 3 or 4 or 5 or 6 or 7 or 8 or 9 or 10 or 11
13. exp body mass/ or exp obesity/ or obese patient/ or morbid obesity/
14. diet therapy/ or exp diet/ or very low calorie diet/ or low calorie diet/
15. body weight loss/ or weight reduction/ or preoperative weight loss.mp.
16. caloric restriction/
17. meal replacement.mp.
18. exp weight loss program/
19. 14 or 15 or 16 or 17 or 18
20. "length of stay"/ or weight complications.mp.
21. surgical complications.mp. or postoperative complication/
22. adverse event/
23. exp treatment outcome/
24. hospital readmission/
25. exp surgical infection/
26. 20 or 21 or 22 or 23 or 24 or 25
27. 12 and 13 and 19 and 26

“elective surgery” OR “orthopedic surgery” OR “revision arthroplasty” OR “total knee arthroplasty” OR “replacement arthroplasty” OR “total arthroplasty” OR “arthroplasty” OR “hip arthroplasty” OR “knee arthroplasty” OR “knee replacement or hip replacement” OR “osteoarthritis” OR “spine surgery” OR “heart surgery” OR “abdominal surgery” OR “laparoscopic cholecystectomy” OR “cholecystectomy” OR “general surgery” OR “surgical patient” AND “body mass” OR “obesity” OR “obese patient” OR “morbid obesity” AND “diet therapy” OR “diet” OR “very low calorie diet” OR “low calorie diet” OR “body weight loss” OR “weight reduction” OR “preoperative weight loss” OR “caloric restriction” OR “meal replacement” OR “weight loss program” AND “length of stay” OR “weight complications” OR “surgical complications” OR “postoperative complication” OR “adverse event” OR “treatment outcome” OR “hospital readmission” OR “surgical infection”

**MeSH terms**

Elective surgery, orthopedic surgery, revision arthroplasty, total knee arthroplasty, replacement arthroplasty, total arthroplasty, hip arthroplasty, knee arthroplasty, arthroplasty, osteoarthritis, spine surgery, heart surgery, abdominal surgery, laparoscopic cholecystectomy, cholecystectomy, general surgery, surgical patient, body mass, obesity, obese patient, morbid obesity, diet therapy, diet, very low calorie diet, low calorie diet, body weight loss, weight reduction, caloric restriction, weight loss program, length of stay, postoperative complication, adverse event, treatment outcome, hospital readmission, surgical infection

**Text words**

Arthroplasty, “$knee replacement or $hip replacement”, preoperative weight loss, meal replacement, weight complications

**Cochrane Database of Systematic Reviews / Cochrane Central Register of Controlled Trials (CENTRAL)**

1. (elective surgery):ti,ab,kw
2. MeSH descriptor: [Elective Surgical Procedures] explode all trees
3. (orthopaedic surgery) :ti,ab,kw
4. MeSH descriptor: [Orthopedic Procedures] explode all trees
5. (arthroplasty) :ti,ab,kw
6. MeSH descriptor: [Arthroplasty] explode all trees
7. MeSH descriptor: [Arthroplasty, Replacement] explode all trees
8. (“knee replacement” OR “hip replacement”) :ti,ab,kw
9. (spinal fusion) :ti,ab,kw
10. MeSH descriptor: [Spinal Fusion] explode all trees
11. MeSH descriptor: [Diskectomy] explode all trees
12. MeSH descriptor: [Laminectomy] explode all trees
13. MeSH descriptor: [Thoracic Surgery] explode all trees
14. MeSH descriptor: [Coronary Artery Bypass] explode all trees
15. (abdominal surgery) :ti,ab,kw
16. MeSH descriptor: [Cholecystectomy] explode all trees
17. MeSH descriptor: [General Surgery] explode all trees
18. (general surgery) :ti,ab,kw
19. #1 OR #2 OR #3 OR #4 OR #5 OR #6 OR #7 OR #8 OR #9 OR #10 OR #11 OR #12 OR #13 OR #14 OR #15 OR #16 OR #17 OR #18
20. (obese or obesity):ti,ab,kw
21. MeSH descriptor: [Obesity] explode all trees
22. MeSH descriptor: [Body Mass Index] explode all trees
23. #20 OR #21 OR #22
24. (diet):ti,ab,kw
25. MeSH descriptor: [Diet] explode all trees
26. (low calorie diet):ti,ab,kw
27. MeSH descriptor: [Caloric Restriction] explode all trees
28. MeSH descriptor: [Weight Loss] explode all trees
29. (weight loss):ti,ab,kw
30. MeSH descriptor: [Weight Reduction Programs] explode all trees
31. (meal replacement):ti,ab,kw
32. #24 OR #25 OR #26 OR #27 OR #28 OR #29 OR #30 OR #31
33. #19 AND #23 AND #32

“elective surgery” OR “elective surgical procedures” OR “orthopaedic surgery” OR “orthopaedic procedures” OR “arthroplasty” OR “arthroplasty, replacement” OR “”knee replacement” or “hip replacement”” OR “spinal fusion” OR “diskectomy” OR “laminectomy” OR “thoracic surgery” OR “coronary artery bypass” OR “abdominal surgery” OR “cholecystectomy” OR “general surgery” AND “obese” OR “obesity” OR “body mass index” AND “diet” OR “low calorie diet” OR “caloric restriction” OR “weight loss” OR “weight reduction programs” OR “meal replacement”

**MeSH terms** (listed above)

**Text words**

Elective surgery, orthopaedic surgery, arthroplasty, “knee replacement” or “hip replacement”, spinal fusion, abdominal surgery, general surgery, obese or obesity, low calorie diet, weight loss, meal replacement

**CINAHL**

**# Query**

S12 S8 AND S9 AND S10 AND S11

S11 S1 OR S2 OR S3 OR S4 OR S5 OR S6 OR S7

S10 (postoperative complications or postoperative issues or postoperative problems) OR (length of stay or hospitalization or length in hospital) OR (hospital readmission, or readmission, or re-hospitalization) OR treatment outcomes

S9 (weight loss or weight reduction or lose weight) OR weight management OR (diet therapy or dietetic therapy or diet) OR (weight loss program or intervention) OR (caloric restriction or low calorie diet) OR meal replacement OR preoperative weight loss

S8 (obese or obesity or overweight) OR morbid obesity OR (body mass index or bmi)

S7 cholecystectomy, laparoscopic OR laparoscopy OR gastrointestinal surgery

S6 abdominal surgery

S5 (cardiac surgery or heart surgery or surgery, cardiovascular or coronary artery bypass or cardiopulmonary bypass) OR (cardiac surgery or heart surgery or cardiothoracic surgery or sternotomy or open cardiac surgery)

S4 (spinal surgery or spine surgery or spinal fusion) OR (laminectomy or discectomy)

S3 (arthroplasty or joint replacement) OR (total knee replacement or total knee arthroplasty) OR (total hip replacement or total hip arthroplasty or hip replacement surgery)

S2 orthopedic surgery

S1 elective surgery

“elective surgery” OR “orthopedic surgery” OR “arthroplasty” OR “joint replacement” OR “total knee replacement” OR “total knee arthroplasty” OR “total hip replacement” OR “total hip arthroplasty” OR “hip replacement surgery” OR “spinal surgery” OR “spine surgery” OR “spinal fusion” OR “laminectomy” OR “discectomy” OR “cardiac surgery” OR “heart surgery” OR “cardiovascular surgery” OR “coronary artery bypass” OR “cardiopulmonary bypass” OR “cardiac surgery” OR “heart surgery” OR “cardiothoracic surgery” OR “sternotomy” OR “open cardiac surgery” OR “abdominal surgery” OR “laparoscopic cholecystectomy” OR “laparoscopy” OR “gastrointestinal surgery” AND “obese” OR “obesity” OR “overweight” OR “morbid obesity” OR “body mass index” OR “BMI” AND “weight loss” OR “weight reduction” OR “lose weight” OR “weight management” OR “diet therapy” OR “dietetic therapy” OR “diet” OR “weight loss program or intervention” OR “caloric restriction” OR “low calorie diet” OR “meal replacement” OR “preoperative weight loss” AND “postoperative complications” OR “postoperative issues” OR “postoperative problems” OR “length of stay” OR “hospitalization” OR “length in hospital” OR “hospital readmission” OR “readmission” OR “re-hospitalization” OR “treatment outcomes”

**MeSH terms**

Elective surgery, orthopedic surgery, “arthroplasty or joint replacement”, “total knee replacement or total knee arthroplasty”, “total hip replacement or total hip arthroplasty or hip replacement surgery”, “spinal surgery or spine surgery or spinal fusion”, “laminectomy or discectomy”, “cardiac surgery or heart surgery or surgery, cardiovascular or coronary artery bypass or cardiopulmonary bypass”, “cardiac surgery or heart surgery or cardiothoracic surgery or sternotomy or open cardiac surgery”, abdominal surgery, laparoscopic cholecystectomy, laparoscopy, gastrointestinal surgery, “obese or obesity or overweight”, morbid obesity, “body mass index or bmi”, “weight loss or weight reduction or lose weight”, weight management, diet therapy, diet, weight loss program or intervention, caloric restriction, low calorie diet, meal replacement, “postoperative complications or postoperative issues or postoperative problems”, “length of stay or hospitalization or length in hospital”, “hospital readmission, or readmission, or re-hospitalization”, treatment outcomes

**Text words**

Dietetic therapy, preoperative weight loss

**Scopus**

TITLE-ABS-KEY ( "postoperative complications"  OR  "surgical complications"  OR  "weight complications"  OR  "length of stay"  OR  "treatment outcomes"  OR  "hospital readmission"  OR  "surgical infection" )

TITLE-ABS-KEY ( diet  OR  "diet therapy"  OR  "weight loss"  OR  "preoperative weight loss"  OR  "weight reduction"  OR  "low calorie diet"  OR  "caloric restriction"  OR  "meal replacement"  OR  "weight loss program" )

TITLE-ABS-KEY ( obese  OR  obesity  OR  "morbid obesity"  OR  "body mass"  OR  "body mass index" )

TITLE-ABS-KEY ( "elective surgery" )  OR  TITLE-ABS-KEY ( "orthopedic surgery" )  OR  TITLE-ABS-KEY ( arthoplasty  OR  "joint replacement"  OR  "total knee arthroplasty"  OR  "total hip arthroplasty" )  OR  TITLE-ABS-KEY ( "spinal surgery"  OR  "spine surgery"  OR  "spinal fusion"  OR  laminectomy  OR  discectomy )  OR  TITLE-ABS-KEY ( "cardiac surgery"  OR  "coronary artery bypass graft"  OR  "valve replacement"  OR  "thoracic surgery" )  OR  TITLE-ABS-KEY ( "abdominal surgery" )  OR  TITLE-ABS-KEY ( cholecystectomy  OR  laparoscopy )  OR  TITLE-ABS-KEY ( "gastrointestinal surgery" )  OR  TITLE-ABS-KEY ( "general surgery" )

“elective surgery” OR “orthopedic surgery” OR “arthroplasty” OR “joint replacement” OR “total knee arthroplasty” OR “total hip arthroplasty” OR “spinal surgery” OR “spine surgery” OR “spinal fusion” OR “laminectomy” OR “discectomy” OR “cardiac surgery” OR “coronary artery bypass graft” OR “valve replacement” OR “thoracic surgery” OR “abdominal surgery” OR “cholecystectomy” OR “laparoscopy” OR “gastrointestinal surgery” OR “general surgery” AND “obese” OR “obesity” OR “morbid obesity” OR “body mass” OR “body mass index” AND “diet” OR “diet therapy” OR “weight loss” OR “preoperative weight loss” OR “weight reduction” OR “low calorie diet” OR “caloric restriction” OR “meal replacement” OR “weight loss program” AND “postoperative complications” OR “surgical complications” OR “weight complications” OR “length of stay” OR “treatment outcomes” OR “hospital readmission” OR “surgical infection”

All terms were used as text words in Scopus.

**Supplementary Table 1. Excluded studies and reasons for exclusion.**

| **Excluded reference** | **Reason for exclusion** |
| --- | --- |
| Pellegrini (2018)^1^ | Criteria was 25-45 kg/m^2^ for recruitment (but baseline BMI was 36.5 ± 5.1kg/m^2^) and the intervention crossed over to postoperative period. |
| Inacio (2014)^2^ | Retrospective study. |
| Gandler (2016)^3^ | Intervention completed in the postoperative period. |
| Grundmann (2018)^4^ | BMI > 35 kg/m^2^ was excluded. Baseline BMI range was 24.7 - 30.7 kg/m^2^. |
| Inacio (2014)^5^ | Retrospective study. |
| Jongbloed (2016)^6^ | Criteria was BMI > 19 kg/m^2^. Baseline BMI was 22-49.9 kg/m^2^. |
| Halbert (2018)^7^ | Study currently in progress. |
| Vo (2019)^8^ | No full text available. |
| Borland (2011)^9^ | Not an RCT (editorial article). |
| Sun (2017)^10^ | Included people with BMI < 30 kg/m^2^. |
| De Luis (2012)^11^ | No control group. |
| ACTRN (2021)^12^ | Study is currently in progress. |
| Ward (2020)^13^ | No full text available. |

**Supplementary Table 2. Postoperative complications.**

| **Outcome** | **Outcome Measure** | **Study** | **Sample size** | | **Acute** | | **From discharge to 30 days post-surgery** | | **Between 30- and 90-days post-surgery** | | **After 90 days post-surgery** | |
| --- | --- | --- | --- | --- | --- | --- | --- | --- | --- | --- | --- | --- |
|  |  |  | **I** | **C** | **I** | **C** | **I** | **C** | **I** | **C** | **I** | **C** |
| **Postoperative complications** | Total number | Burnand (2016) | 21 | 25 | 0 (0) | 1 (4) | 1 (4.8) | 1 (4) | - | - | - | - |
|  |  | Hollis (2020) | 20 | 14 | 0 (0) | 0 (0) | 0 (0) | 2 (14.2) | - | - | - | - |
|  |  | Liljensøe (2019) | 38 | 38 | 1 (2.6) | 2 (5.3) | 0 (0) | 0 (0) | 0 (0) | 0 (0) | 2 (5.3) | 3 (7.9) |
| **Wound complication** | Surgical wound complication  (not infection) | Liljensøe (2019) | 38 | 38 | 0 (0) | 1 (2.6) | - | - | - | - | - | - |
|  | Secretion from wound on day 0 |  |  |  | 1 (2.6) | 6 (15.8) | - | - | - | - | - | - |
|  | Wound infection | Hollis (2020) | 20 | 14 | - | - | 0 (0) | 2 (14.2) | - | - | - | - |
|  |  | Liljensøe (2019) | 38 | 38 | - | - | - | - | - | - | 1 (2.6) | 1 (2.6) |
| **Infection**  **(unrelated to wound)** | Urinary tract infection | Liljensøe (2019) | 38 | 38 | 0 (0) | 1 (2.6) | - | - | - | - | - | - |
| **Change of procedure** | Conversion during surgery | Burnand (2016) | 21 | 25 | 0 (0) | 1 (4) | - | - | - | - | - | - |
|  | Revision surgery | Liljensøe (2019) | 38 | 38 | - | - | - | - | - | - | 1 (2.6) | 1 (2.6) |
| **Arthroplasty related complications** | Manipulation under anaesthetic | Liljensøe (2019) | 38 | 38 | - | - | - | - | - | - | 1 (2.6) | 1 (2.6) |
|  | Dislocation |  |  |  | - | - | - | - | - | - | 0 (0) | 1 (2.6) |
| **Other complications** | Quadriceps microfiber blasts | Liljensøe (2019) | 38 | 38 | 1 (2.6) | 0 (0) | - | - | - | - | - | - |
| **Length of stay** | Hours    Days | Burnand (2016) | 21 | 25 | 10.52 (5.94) | 13.75 (27.62) | - | - | - | - | - | - |
|  |  | Hollis (2020) | 20 | 14 | 1.2 (0.5) | 1.36 (0.7) | - | - | - | - | - | - |
|  |  | Liljensøe (2019) | 38 | 38 | 3.1 (1.5) | 3.3 (2.1) | - | - | - | - | - | - |
| **Readmission related to surgery** | Number | Burnand (2016) | 21 | 25 | - | - | 1 (4.8) | 1 (4) | 0 (0) | 0 (0) | 0 (0) | 0 (0) |
|  |  | Hollis (2020) | 20 | 14 | - | - | 0 (0) | 0 (0) | 0 (0) | 0 (0) | 0 (0) | 0 (0) |
|  |  | Liljensøe (2019) | 38 | 38 | - | - | 0 (0) | 0 (0) | 0 (0) | 0 (0) | 0 (0) | 0 (0) |

Outcomes are expressed as number of events (%) except for length of stay data which is expressed as mean (standard deviation). I: Intervention; C: Control.

Note: both revision surgeries occurring after 90 days post-surgery in Liljensøe et al’s study are due to the wound infections. As such, the wound infection and subsequent revision surgery are counted as one complication.

**Supplementary Table 3. Outcome data.**

| **Outcome** | **Outcome Measure** | **Study** | **Sample size** | | **Baseline** | | **Baseline to pre-surgery** | | **Baseline to one year** | |
| --- | --- | --- | --- | --- | --- | --- | --- | --- | --- | --- |
|  |  |  | **I** | **C** | **I** | **C** | **I** | **C** | **I** | **C** |
| **Weight change** | kg | Burnand (2016) | 21 | 25 | - | - | -3.48 (1.98) | -0.98 (1.67) | - | - |
|  |  | Hollis (2020) | 23 | 14 | - | - | -6.5 (3.8) | 0.1 (2.6) | - | - |
|  |  | Liljensøe (2019) | 38 | 38 | - | - | -10.7 (-11.7- [-9.6])^a^ | 0.3 (-1.1-1.6)^a^ | -9.6 (-12.0-7.2)^a^ | 0.6 (-0.9-2.0)^a^ |
|  | % | Burnand (2016) | 21 | 25 | - | - | -3.72 (2.3) | -1.05 (1.7) | - | - |
|  |  | Hollis (2020) | 23 | 14 | - | - | -5.5 (3.47) | 0.11(2.4) | - | - |
|  |  | Liljensøe (2019) | 38 | 38 | - | - | -10.09 (2.6) | -0.14 (3.9) | - | - |
|  | BMI (kg/m^2^) | Burnand (2016) | 21 | 25 | - | - | -1.29 (0.74) | -0.36 (0.61) | - | - |
|  |  | Hollis (2020) | 23 | 14 | - | - | - | - | - | - |
|  |  | Liljensøe (2019) | 38 | 38 | - | - | -3.2 (0.8) | -0.05 (1.2) | -2.87 (2.2) | 0.15 (1.3) |
| **PROM** | SF-36 PCS | Liljensøe (2019) | 38 | 38 | 37.9 (36.2-39.5)^a^ | 39.8 (37.8-41.8)^a^ | - | - | 7.7 (5.3-10.04)^a^ | 7.8 (5.0-10.6)^a^ |
|  | SF-36 MCS |  |  |  | 42.8 (39.2-46.4)^a^ | 48.9 (45.2-52.6)^a^ | - | - | 4.9 (1.0-8.9)^a^ | 5.6 (3.1-8.1)^a^ |
|  | KOOS ADL |  |  |  | 45.9 (39.3-52.5)^a^ | 52.1 (47.3-59.9)^a^ | - | - | 25.9 (18.8-32.9)^a^ | 25.9 (19.1-32.1)^a^ |
|  | KOOS QOL |  |  |  | 23.2 (18.7-27.6)^a^ | 32.9 (28.3-37.5)^a^ | - | - | 31.3 (23.9-38.6)^a^ | 32.9 (24.0-41.8)^a^ |
|  | KOOS Symptoms |  |  |  | 50.0 (45.8-56.1)^a^ | 53.1 (47.3-58.9)^a^ | - | - | 19.0 (11.6-26.4)^a^ | 22.1 (16.6-27.6)^a^ |
|  | KOOS Pain |  |  |  | 40.9 (35.6-46.2)^a^ | 48.5 (43.3-53.8)^a^ | - | - | 33.1 (25.9-40.3)^a^ | 29.8 (22.7-36.9)^a^ |
|  | KOOS Sport/Rec. |  |  |  | 10.4 (5.9-14.9)^a^ | 11.8 (6.5-17.2)^a^ | - | - | 19.2 (12.8-25.6)^a^ | 24.8 (15.1-33.8)^a^ |
|  | IWQOL-Lite | Hollis (2020) | 15 | 4 | 60.8 (15.9)^b^ | 67.1 (23.9)^c^ | 18  (6-41.4)^d^ | 4.6  (-14-13.85)^d^ | - | - |

Outcomes are expressed as mean (standard deviation) unless otherwise specified. I: Intervention; C: Control; BMI: Body Mass Index; SF-36: short-form 36; PCS: Physical Component Score; MCS: Mental Component Score; KOOS: Knee injury and Osteoarthritis Outcome Score; ADL: Activities of Daily Living; QoL: quality of life; Sport/Rec.: Sport and Recreation; IWQOL-Lite: Impact of Weight on Quality of Life-Lite.

1. Mean difference and 95% CI
2. n = 21 completed
3. n = 19 completed
4. Median (range)

1. Pellegrini CA, Chang RW, Dunlop DD, et al. Comparison of a Patient-Centered Weight Loss Program starting before versus after knee replacement: A pilot study. *Obes Res Clin Pract.* 2018;12(5):472-478.

2. Inacio MC, Kritz-Silverstein D, Raman R, et al. The impact of pre-operative weight loss on incidence of surgical site infection and readmission rates after total joint arthroplasty. *Journal of Arthroplasty.* 2014;29(3):458-464.e451.

3. Gandler N, Simmance N, Keenan J, Choong PF, Dowsey MM. A pilot study investigating dietetic weight loss interventions and 12 month functional outcomes of patients undergoing total joint replacement. *Obes Res Clin Pract.* 2016;10(2):220-223.

4. Grundmann F, Muller RU, Reppenhorst A, et al. Preoperative short-term calorie restriction for prevention of acute kidney injury after cardiac surgery: A randomized, controlled, open-label, pilot trial. *Journal of the American Heart Association.* 2018;7(6).

5. Inacio MCS, Kritz-Silverstein D, Raman R, et al. The risk of surgical site infection and readmission in obese patients undergoing total joint replacement who lose weight before surgery and keep it off post-operatively. *Bone and Joint Journal.* 2014;96 B(5):629-635.

6. Jongbloed F, de Bruin RW, Klaassen RA, et al. Short-Term Preoperative Calorie and Protein Restriction Is Feasible in Healthy Kidney Donors and Morbidly Obese Patients Scheduled for Surgery. *Nutrients.* 2016;8(5):20.

7. Nct. Utilization of Very Low Calorie Diet in Obese General Surgery Patients. [*https://clinicaltrialsgov/show/NCT03553849*](https://clinicaltrialsgov/show/NCT03553849)*.* 2018.

8. Vo T, Marney L, Mundy J, Edney G, Griffin R. Very Low-Calorie Diet in Cardiac Surgery Patients With Obesity and Metabolic Syndrome. *Heart Lung and Circulation.* 2019;28 (Supplement 3):S114-S115.

9. Borland WS, Jennings AG. Weight loss for obese patients prior to total knee replacement. *Journal of Clinical Orthopaedics and Trauma.* 2011;2(2):127.

10. Sun BJ, Valdez D, Duong D, Gupta R, Smith BR. Evaluation of preoperativeweight loss for elective hernia repair in the veteran population. *American Surgeon.* 2017;83(10):1112-1116.

11. de Luis DA, Izaola O, García Alonso M, Aller R, Cabezas G, de la Fuente B. Effect of a commercial hypocaloric diet in weight loss and post surgical morbidities in obese patients with chronic arthropathy, a randomized clinical trial. *European review for medical and pharmacological sciences.* 2012;16(13):1814-1820.

12. Actrn. Does preoperative dietitian-led Very Low Calorie Diet (VLCD)-based intervention reduce unfavourable elective surgery outcomes for patients with obesity? [*http://wwwwhoint/trialsearch/Trial2aspx?TrialID=ACTRN12621000084886*](http://wwwwhoint/trialsearch/Trial2aspx?TrialID=ACTRN12621000084886)*.* 2021.

13. Ward R, Mori, K. Role of pre-operative Very Low-Calorie Diet (VCLD) in obese general surgery patients. Northern Health Research Week; 2020; Epping, Australia.

**REFERENCES**
